# Imaging spectroscopy reveals the effects of topography and logging on the leaf chemistry of tropical forest canopy trees

**DOI:** 10.1111/gcb.14903

**Published:** 2019-12-17

**Authors:** Tom Swinfield, Sabine Both, Terhi Riutta, Boris Bongalov, Dafydd Elias, Noreen Majalap‐Lee, Nicholas Ostle, Martin Svátek, Jakub Kvasnica, David Milodowski, Tommaso Jucker, Robert M. Ewers, Yi Zhang, David Johnson, Yit Arn Teh, David F. R. P. Burslem, Yadvinder Malhi, David Coomes

**Affiliations:** ^1^ Forest Ecology and Conservation Group Department of Plant Sciences University of Cambridge Cambridge UK; ^2^ Centre for Conservation Science Royal Society for the Protection of Birds Cambridge UK; ^3^ School of Biological Sciences University of Aberdeen Aberdeen UK; ^4^ Environmental and Rural Science University of New England Armidale NSW Australia; ^5^ Environmental Change Institute School of Geography and the Environment University of Oxford Oxford UK; ^6^ Centre for Ecology & Hydrology Lancaster Environment Centre Lancaster UK; ^7^ Lancaster Environment Centre Lancaster University Lancaster UK; ^8^ Sabah Forestry Department Forest Research Centre Sandakan Malaysia; ^9^ Department of Forest Botany, Dendrology and Geobiocoenology Faculty of Forestry and Wood Technology Mendel University in Brno Brno Czech Republic; ^10^ School of GeoSciences University of Edinburgh Edinburgh UK; ^11^ National Centre for Earth Observation University of Edinburgh Edinburgh UK; ^12^ School of Biological Sciences University of Bristol Bristol UK; ^13^ Imperial College London Ascot UK; ^14^ School of Earth and Environmental Sciences The University of Manchester Manchester UK

**Keywords:** imaging spectroscopy, leaf traits, logging, nutrient availability, phosphorus, specific leaf area, topography, tropical forest

## Abstract

Logging, pervasive across the lowland tropics, affects millions of hectares of forest, yet its influence on nutrient cycling remains poorly understood. One hypothesis is that logging influences phosphorus (P) cycling, because this scarce nutrient is removed in extracted timber and eroded soil, leading to shifts in ecosystem functioning and community composition. However, testing this is challenging because P varies within landscapes as a function of geology, topography and climate. Superimposed upon these trends are compositional changes in logged forests, with species with more acquisitive traits, characterized by higher foliar P concentrations, more dominant. It is difficult to resolve these patterns using traditional field approaches alone. Here, we use airborne light detection and ranging‐guided hyperspectral imagery to map foliar nutrient (i.e. P, nitrogen [N]) concentrations, calibrated using field measured traits, over 400 km^2^ of northeastern Borneo, including a landscape‐level disturbance gradient spanning old‐growth to repeatedly logged forests. The maps reveal that canopy foliar P and N concentrations decrease with elevation. These relationships were not identified using traditional field measurements of leaf and soil nutrients. After controlling for topography, canopy foliar nutrient concentrations were lower in logged forest than in old‐growth areas, reflecting decreased nutrient availability. However, foliar nutrient concentrations and specific leaf area were greatest in relatively short patches in logged areas, reflecting a shift in composition to pioneer species with acquisitive traits. N:P ratio increased in logged forest, suggesting reduced soil P availability through disturbance. Through the first landscape scale assessment of how functional leaf traits change in response to logging, we find that differences from old‐growth forest become more pronounced as logged forests increase in stature over time, suggesting exacerbated phosphorus limitation as forests recover.

## Graphical abstract

Soil nutrients that come from rocks are critical for tree growth but rare in tropical forests. Logging removes these nutrients in quantities that could change plant life there. We mapped leaf nutrients in pristine and logged forests in Malaysia using cutting edge remote sensing combined with field measurements and found fewer nutrients in logged forests after controlling for other variables. Nutrients were most scarce in logged forests that had recovered with time after logging, raising concerns that they are changed permanently in terms of the species that live there and the nutrients available to them.

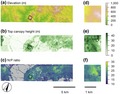

## 1 INTRODUCTION

Lowland tropical forests have been logged extensively, yet the influence of timber extraction on nutrient cycling remains poorly understood. Timber extraction has produced vast swathes of degraded tropical forests, which are now more widespread than primary forests (Laurance, Sayer, & Cassman, 2014). Even in their degraded state, rainforests retain the majority of their species and have the potential to capture huge quantities of atmospheric carbon if allowed to regrow (Edwards, Tobias, Sheil, Meijaard, & Laurance, 2014; Poorter et al., 2016). However, logging removes mineral nutrients through exported logs, leaching and soil erosion as well as gaseous emissions (Cleveland, Reed, & Townsend, 2006; Quinton, Govers, Oost, & Bardgett, 2010), raising concerns about the long‐term sustainability of logging in tropics (Imai, Kitayama, & Titin, 2012).

The biogeochemistry of nitrogen (N) and phosphorus (P) is affected by soil age, topography and elevation and is relatively well understood. Rock‐derived elements, such as P, are generally considered to limit plant growth in old, heavily weathered tropical soils because they are depleted due to millions of years of weathering and erosion (Walker & Syers, 1976), while much that remains is sequestered in stable aluminium (Al) compounds that are resistant to mineralization (Crews, 2016; Heineman, Turner, & Dalling, 2016; McGroddy, Daufresne, & Hedin, 2004; Tanner, Vitousek, & Cuevas, 1998). Although the total pool size of P varies widely between soil types, due to soil age and differences in parent material chemical composition (Chadwick & Asner, 2016), its availability is also influenced by topography, because landscape position influences rates of weathering, erosion and deposition (Chadwick & Asner, 2016; Richardson, Allen, & Doherty, 2008). Nitrogen (N) is fixed by microbes, and tends to accumulate over the course of primary succession, so is not typically limiting in lowland rainforests on highly weathered soils (Nasto et al., 2014). However, N availability is often lower at higher altitudes, probably as a result of climatological controls on the abundance of N fixing species (Soethe, Lehmann, & Engels, 2008; Steidinger et al., 2019). These differences in soil nutrient availability are reflected in the positioning of foliar traits along the leaf economics spectrum (Díaz et al., 2016; Wright et al., 2004) with nutrient rich soils filtering for traits associated with fast growth and rapid acquisition of resources (e.g. low leaf mass per area, high foliar N and P concentrations and low wood density) while nutrient‐poor soils filter for conservative traits that confer survival (e.g. tough leaves containing high concentrations of secondary metabolites and dense wood).

However, the effect of logging on biogeochemical cycling and the consequences for ecosystem functions, such as net primary productivity, are less well understood. On the one hand, logged forests undergo rapid secondary succession, accumulating up to 90% of pre‐disturbance biomass within 66 years in the neotropics (Poorter et al., 2016). One explanation for this apparent lack of negative impacts is that surviving understory trees and recolonizing plants take advantage of the pulse of nutrients released by decomposing roots, bark, fine branches and leaves left in situ (Denslow, Ellison, & Sanford, 1998), partially or wholly mitigating any nutrient losses caused by biomass removal. Furthermore, the reduced top‐of‐canopy height (TCH) and lower density of large trees, leads to decreases in both above‐ and below‐ground competition, increasing resource availability and thus the dominance of resource‐acquisitive traits (Baraloto et al., 2012; Carreño‐Rocabado et al., 2012), fueling recolonization and growth (Teh, Silver, & Scatena, 2009). Early‐successional species with resource‐acquisitive traits may persist in logged forests for many decades (Baraloto et al., 2012; Carreño‐Rocabado et al., 2016), but resource‐conservative traits are expected to again dominate once mineral resources become limited with increased above‐ground biomass, canopy closure and root competition (Coomes & Grubb, 2000). However, if nutrient limitation is exacerbated by repeat rounds of logging, forests may shift to even more resource‐conservative states than old‐growth forests (Carreño‐Rocabado et al., 2016), with foliar N:P ratios elevated beyond the region within which N and P are thought to be co‐limiting (14–16; Koerselman & Meuleman, 1996), which could affect plant productivity (McGroddy et al., 2004; Porder, Vitousek, Chadwick, Chamberlain, & Hilley, 2007; Turner, Brenes‐Arguedas, & Condit, 2018; Zalamea et al., 2016).

The Sustainability of Altered Forest Ecosystems (SAFE) experiment in Sabah, Malaysia (Ewers et al., 2011), provides an outstanding location to evaluate the impacts of logging on biogeochemical cycles. An extensive field campaign was undertaken to investigate how ecosystem processes and functional traits changed in response to different intensities of logging (Both et al., 2019; Riutta et al., 2018). Logged forests were found to be 41% more productive than old‐growth forests once differences in basal area were taken into account (Riutta et al., 2018), because fast‐growing pioneer species grow rapidly in response to elevated resources (Brokaw, 1985; Gustafsson et al., 2016; Slik, 2005; Verburg & van Eijk‐Bos, 2003). Both et al. (2019) identified that logged forest communities were dominated by species with acquisitive traits, while old‐growth areas were dominated by species with conservative traits; similar trends in leaf and wood economics have been reported in other tropical forests (Baraloto et al., 2012; Carreño‐Rocabado et al., 2012, 2016). However, with only small numbers of independent field measurements, it was not possible to separate the role of logging from that of landscape scale nutrient distributions.

Airborne imaging spectrometry (also known as ‘spectranomics’; Asner & Martin, 2009) makes it possible to study the spatial distribution of foliar plant traits at broad spatial scales, enabling the effects of logging to be unpicked from the natural background variability of leaf properties. This approach provides an unrivalled tool for scaling‐up point‐based measurements of ecosystem processes that would otherwise be impossible with traditional field measurements alone (e.g. Asner, Anderson, et al., 2015; Asner & Martin, 2009, 2016a, 2016b; Chadwick & Asner, 2016; Ollinger et al., 2002; Schneider et al., 2017). By measuring electromagnetic radiation, reflected by land surfaces, in hundreds of narrow wavebands in the visible, near‐ and shortwave infrared, the distinct chemical and physical properties of forest canopies can be detected (Asner, Martin, Anderson, & Knapp, 2015; Doughty et al., 2017; Nunes, Davey, & Coomes, 2017). The spectranomic approach has been used to measure foliar nutrient concentrations and describe patterns of biogeochemical cycling (e.g. Asner, Martin, et al., 2015; Schneider et al., 2017), and has identified the importance of topographic processes in shaping ecosystem function by modulating canopy structure and species richness (Jucker et al., 2018). Furthermore, it has been used to demonstrate changes in foliar traits and canopy function in response to anthropogenic disturbance (Ollinger et al., 2002) and invasions by non‐native species (Balzotti & Asner, 2018). Yet, as far as we are aware no such studies have yet attempted to assess the effect of logging on canopy function while accounting for background variation in nutrient availability.

This study used airborne imaging spectroscopy in conjunction with the detailed trait assessment of Both et al. (2019) to assess the effect of logging on canopy foliar traits. Using cross‐validated partial least squares regression (PLSR), we developed models to estimate foliar traits from spectral observations and used these models to predict foliar traits over 400 km^2^ of old‐growth and heterogeneously logged tropical forest in the Malaysian state of Sabah, on the island of Borneo. Maps of foliar N and P concentration (concentrations henceforth indicated with square brackets), N:P ratios and specific leaf area (SLA) were produced to assess the relative importance of logging when compared with natural trait expression. Light detection and ranging (LiDAR)‐derived measurements of elevation and TCH were used to describe landscape patterns in topography, logging intensity and regeneration. The relationships were also compared with field measured community‐weighted mean traits and soil nutrient pools. The extent to which canopy foliar traits differed between logged and old‐growth forests was assessed while holding environmental variables constant. Our hypotheses were that:

1. Foliar [N] and [P] and SLA are highest in low elevation sites within the hilly (i.e. topographically diverse) landscape, reflecting the underlying distributions of soil nutrient availability driven by erosional and depositional processes;
2. Foliar [N] and [P] and SLA are elevated in short forest patches in both logged and old‐growth forests, reflecting transitions in composition towards species with resource‐acquisitive traits;
3. After controlling for elevation and canopy height, foliar nutrient concentrations are lower and SLA is higher in logged forest than in old‐growth forest due to lower nutrient availability;
4. P becomes increasingly limiting with elevation and logging, exhibited as increased foliar N:P ratios.

## 2 MATERIALS AND METHODS

### 2.1 Study site

Our study was located in three regions of Sabah, a Malaysian state in northern Borneo: old‐growth forests in the Maliau Basin and Danum Valley Conservation Areas, and logged forest fragments in the Kalabakan Reserve, within which the Stability of Altered Forest Ecosystems project is located (SAFE, http://www.safeproject.net; see also Ewers et al., 2011). Hereafter, the forest areas are referred to as Maliau, Danum and SAFE respectively. Although some low intensity selective logging occurred at Maliau and Danum in the 1970s, the majority of these sites have not been disturbed and are dominated by tall old‐growth dipterocarp forest and are therefore referred to as *old‐growth* forest. Almost all of SAFE has been selectively logged once or twice, first in the 1970s and then the 1990s, with some places logged four times, and is therefore referred to generically as *logged*. Much of SAFE was actively being converted during the study through salvage logging and forest clearance to oil palm (Ewers et al., 2011). Therefore, our study site contained a forest disturbance gradient from primary to recently cleared forest.

The areas varied in terms of elevation, topography, soils and forest structure. Four predominant soil formations were present. SAFE and Danum are underlain by the Bang (orthic acrisols, dystric cambisols), Gumpal (orthic acrisols, orthic luvisols, dystric/ eutric cambisols, lithosols) and Mentapuk (chromic/orthic luvisols, eutric cambisols, lithosols) soils formations, while Maliau occupied the Maliau soil type (orthic acrisols, dystric cambisols, gleyic podzols, humic gleysols, lithosols; Acres et al., 1975). Fluvial incision within the Maliau study area has generated a river valley descending from 850 to 250 m a.s.l.; Danum was lower lying and relatively flat, with elevation ranging between 200 and 400 m a.s.l.; and SAFE has a varied topography with the lowlands (100–350 m a.s.l.) almost entirely converted to oil palm and the remaining forest predominantly covering hills rising to over 1,000 m a.s.l.

Eight 1 ha plots were located in these landscapes, as part of the pantropical Global Ecosystems Monitoring network (http://gem.tropicalforests.ox.ac.uk/). The plots spanned the gradient of logging intensity across the forest areas from pristine old‐growth forest to heavily logged forests (Ewers et al., 2011). Maliau and Danum both had two plots each and the remaining four were located within logged forest fragments at SAFE, two were ‘moderately logged’ (i.e. selectively logged twice) and two were ‘heavily logged’ (i.e. selectively logged four times; Riutta et al., 2018). Soil and leaf nutrient sampling was conducted in these plots as reported in Both et al. (2019) and presented in Supporting Information S3 and S4.

### 2.2 Airborne campaign

An airborne survey was undertaken by the Natural Environmental Research Council (NERC) Airborne Research Facility (ARF) in November 2014 capturing both LiDAR and hyperspectral data. A Dornier 228‐201 was flown at an altitude of 1,400–2,400 m a.s.l. (depending on the study site) with a flight speed of 120–140 knots with a ground‐based Leica base station running simultaneously to permit sub‐meter accuracy georeferencing of all data.

Light detection and ranging data were collected using a Leica ALS50‐II LiDAR sensor, which emits pulses at a frequency of 120 kHz, has a field of view of 12° and a footprint of approximately 40 cm. Preprocessing of the LiDAR data was undertaken by NERC's Data Analysis Node and delivered as discrete returns (full details of the processing workflow can be found at https://nerc-arf-dan.pml.ac.uk/trac/wiki/procedures). Subsequent point cloud processing was undertaken using LAStools (http://rapidlasso.com/lastools). The point cloud was classified to ground and non‐ground returns using *lasground* with step size set to 10 m, the former were used to produce a triangular irregular network digital terrain model gridded at 0.5 m resolution. All return heights were then normalized to produce a pit free canopy height model by subtraction of ground elevations, following Khosravipour, Skidmore, Isenburg, Wang, and Hussin (2014).

Hyperspectral data were collected using an AisaFENIX sensor which uses two parallel spectrometers to collect continuous intensity measurements from the blue to short wave infrared (380–2,500 nm). Spatial resolution varied between 1 and 4.5 m depending on survey altitude. At the lowest altitudes, the spectral resolution (i.e. the number of discrete spectral bands) was reduced from an average of 6–12 nm during data collection to maximize signal to noise ratio at the sensor. Raw spectra were radiometrically calibrated to irradiance by the NERC ARF using laboratory collected informed radiometric calibration coefficients and spectral calibration. Signal to noise ratio was enhanced by averaging every three bands in the visible and the near‐infrared regions (420–1,100 nm) and every two bands in the shortwave infrared region (1,100–2,400 nm); this took advantage of the strong correlation between adjacent bands to smooth out random noise, from sources such as electrical interference, making the general reflectance pattern more robust but possibly obscuring narrow absorption features. Images from each flight were then georeferenced to the LiDAR‐derived digital elevation model generated from the same flight. Atmospheric and Bidirectional Reflectance Distribution Function correction was applied with the rugged terrain algorithm in the Modtran radiative transfer model in ATCOR v. 6.3.2 which accounted for the solar and viewing angles of each pixel during flight to calculate ground reflectance. Spectra were then brightness normalized following the approach of Feilhauer, Asner, Martin, and Schmidtlein (2010).

### 2.3 Foliar trait measurements

**Figure 1:**
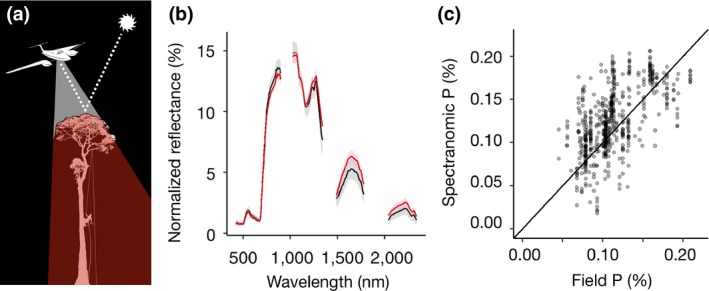
Airborne imaging spectroscopy. (a) A plane flies over the forest canopy simultaneously collecting light detection and ranging (LiDAR) and imaging spectroscopy data. LiDAR uses a scanning laser to measure the three‐dimensional surfaces of the forest canopy and underlying topography. The intensity of sunlight reflected from illuminated leaves is measured in hundreds of continuous bands*,* each covering 6–12 nm, from 380 to 2,500 nm. Sunlit leaves are collected from the same tree canopies by climbers, which are used to measure leaf traits, including foliar nutrient concentrations. (b) Leaves with different chemical and physical properties have different reflectance spectra. (c) Cross‐validated partial least squares regression is used to produce ‘spectranomic’ predictions of field measured canopy traits from the spectra measured for the same tree crowns from which leaves were collected

Foliar traits of 651 individual trees from 284 species were measured during a field campaign undertaken between July and December 2015 (see Both et al., 2019 for more details). A community‐focused sampling design was implemented at the 1 ha global ecosystem monitoring (GEM) plot level, whereby species were ranked by their contribution to total basal area (for all stems ≥10 cm diameter at breast height; DBH) and sampled from greatest to least contribution, until 70% of the plot basal area was sampled. Most species occurred individually but when multiple individuals were present, only the largest was sampled. This was supplemented by sampling all trees ≥10 cm DBH within three 20 m × 20 m subplots, selected at random, within each 1 ha plot. Together these approaches ensured thorough sampling at the community level, with more than 90% of the basal area in seven of the eight plots, and 51%–71% of the species (≥10 cm DBH) sampled. Leaves were collected from fully sunlit branches approximately 2–4 cm in diameter by tree climbing or using telescopic pruners, and undamaged mature leaves were selected and cleaned with water for subsequent analyses (Figure 1). SLA was measured at the field laboratory. Dried bulked and milled leaf material was used for determination of [P] by flow injection analysis after sulphuric acid and hydrogen peroxide digestion and [N] by Kjeldahl analysis, following standardized protocols (Pérez‐Harguindeguy et al., 2013). These were also used to calculate community‐weighted mean foliar traits as described in Supporting Information S3.

Within each of the eight GEM plots DBH, total tree height, *xyz* position within the plot and the spatial extent of crowns for all trees ≥10 cm DBH were recorded between 20 September and 12 October 2016. Stem and crown positions were measured with decimetre spatial precision following the ground‐based laser method (Field‐Map technology, IFER, Czech Republic, https://www.fieldmap.cz/) described in Hédl et al. (2009). Between 5 and 30 points were recorded for each crown and smoothed spatial polygons were fitted to these. The crown polygons were then georeferenced to the plot origins geolocated using a Geneq SXBlue II GPS unit, which uses satellite‐based augmentation to perform differential correction and provides subcanopy positional accuracy of less than 1 m.

### 2.4 Predicting foliar traits from hyperspectral imagery

Tree level spectra, measured from the aircraft, were matched with the traits measured on the same trees in the field. This was achieved by manually aligning the field measured tree crown polygons to the LiDAR CHM using QGIS (version 2.18). Any trees that could not be aligned with high confidence, including those entirely occluded by overstorey crowns, were omitted (see Supporting Information S1). All hyperspectral pixels (spectra) sampled from vegetation shorter than 4 m, as measured by the LiDAR canopy height model, were filtered to exclude non‐tree vegetation. Pixels with NDVI ≥ 0.75 and solar zenith angles at measurement greater than 50° were extracted if ≥80% of the pixel fell within the crown polygons; this value was chosen to provide the best trade‐off between data purity and quantity, and enabled data for some of the smallest trees to be used. Low resolution (12 nm bandwidth) spectra were resampled to match high resolution (6 nm bandwidth) spectra, which made up the majority of the hyperspectral data, through linear interpolation using the *splines* package (version 3.4.0) in R. These were then trimmed following the method of Asner, Martin, et al. (2015) at the far ends of the two spectrometers (<420 nm, >2,400 nm and between 900 and 1,000 nm) and at water absorption bands (1,350–1,480 nm and 1780–2032 nm; Figure 1). The filtering process is necessarily strict and ensures high data quality. The leaf traits of the 104 trees remaining after filtering showed considerable variation at the plot level and were representative of both the mean and overall trait distributions observed. A detailed comparison of the trait distributions for both the full set of trees surveys and those used in building the predictive models is presented in Supporting Information S2. By taking an individual tree approach, we were able to use the full variation in the traits and spectra within the plots.

Partial least squares regression was used to predict foliar traits from 714 spectral observations of 104 unique trees; this chemometric approach has been demonstrated repeatedly to produce accurate predictions of traits from imaging spectroscopy (Asner & Martin, 2016a; Asner, Martin, et al., 2015; Chadwick & Asner, 2016). We included repeat spectral observations (from multiple flights over the plots) individually, rather than averaging a priori, because we considered this to produce more realistic estimates of model accuracy when predicting to new flight lines that may differ, however subtly, in terms of atmospheric and illumination conditions (Gao, Montes, Davis, & Goetz, 2009). The predictive performance of the PLSR models was assessed using two methods: (a) by predicting the trait values of individual pixels (leave‐one‐pixel‐out [LOPO]), which has previously been used in spectranomic assessments at the individual tree scale (Chadwick & Asner, 2018, 2016) and is likely to generate inflated model performance metrics; (b) predicting the trait values of individual trees withheld during model development (leave‐one‐tree‐out [LOTO]) generates a more conservative model performance metric because it explicitly measures uncertainty arising from spectral variation between tree crown pixels resulting from differences in reflectance, spectral mixing and traits. Cross‐validation was implemented in the *autopls* package (version 1.3) in R. The difference from the field measured values was assessed using root mean squared error (RMSE) and *R* ^2^ of the predictions. RMSE values were calculated as:

(1) $$\text{RMSE}=\sqrt{\sum_{i=1}^{n}{\left(Y_{i}-{\hat{Y}}_{i}\right)}^{2}},$$

where $\hat{Y}$ is the vector of trait predictions generated by PLSR cross‐validation for the *n* pixels/trees and *Y* is the vector of observed trait values. These were then converted to percentages of the average trait value ($\bar{Y}$). *R* ^2^ values were calculated as:

(2) $$R^{2}=\left(1-\frac{\sum_{i=1}^{n}{\left(Y_{i}-{\hat{Y}}_{i}\right)}^{2}}{\sum_{i=1}^{n}{\left(Y_{i}-\bar{Y}\right)}^{2}}\right)\times 100.$$

To avoid overfitting the number of orthogonal spectral weighting components used was assessed using the standard method for PLRS, by choosing the model with the fewest components that generated predictions less than one standard error away from the overall best model (Mevik & Wehrens, 2007).

Full coverage maps of foliar traits were produced by making predictions for each flight line from the PLSR models developed from the field measured traits. LOTO models were used for all traits, except SLA for which the, likely over‐fitted, LOPO model was used. The flight line predictions were then mosaicked by averaging to produce site‐level rasters for each trait at 1 ha resolution.

### 2.5 Modelling landscape‐scale variation in foliar stoichiometry

Simultaneous autoregressive multiple regression was used to model the effects of topography, TCH and disturbance history (old‐growth vs. logged) on foliar traits predicted by imaging spectroscopy, while accounting for spatial autocorrelation within locally similar neighbourhoods (see below), using the *spdep* package (version 0.7‐7) in R. Average elevation in metres above sea level (m a.s.l.) at 1 ha scale was used to describe the relative topographic position within the landscape and was extracted from the LiDAR‐derived digital terrain model with higher and lower values indicating uphill and downhill positions, respectively; topographic position index, calculated at the 1, 2.25 and 3.8 ha scales, was also tested but fits the data less well than elevation. TCH is widely used to assess forest stature and maturity because it is closely correlated with above‐ground biomass and can readily be measured using LiDAR (Asner & Mascaro, 2014). Furthermore, because all large stems of commercial value were removed during logging in Sabah (Fisher, Edwards, Giam, & Wilcove, 2011) TCH gives a good indication of disturbance intensity with far greater TCH in old‐growth forest compared with logged forests. Short forest stands with TCH values 4–20 m tall are likely to be young and recovering, while tall forest stands with TCH values greater than 20 m are likely to be mature. By making comparisons between the trait values in old‐growth and logged forests at a given TCH, we are therefore able to assess the effect of logging on canopy stoichiometry and functioning.

Trait maps were spatially autocorrelated which needed to be accommodated in statistical modelling. We first estimated spatial autocorrelation by fitting anisotropic semivariograms (R package *gstat*) with a 4 km maximum range to the residuals produced by ordinary least‐squares regression. We then used simultaneous autoregressive multiple regression (R package *spdep*) with the extent of the first‐order neighbourhood determined as 2 km by the semivariograms. Trait values were modelled as linear functions of the interactions between disturbance history (logged vs. old‐growth), elevation and TCH to evaluate whether logging affected foliar traits after accounting for elevation. TCH and elevation were scaled and centred at 30 m tall forest at 200 m a.s.l. prior to analysis in order to use 30 m tall old‐growth forest at low elevation as the reference. A bootstrapping approach was used to estimate model parameters and their confidence intervals, with models fitted separately to 1,000 random samples of 1,000 pixels. 95% confidence intervals were calculated according to the 2.5th and 97.5th percentiles of the estimated parameters and difference from zero within this range was used as the threshold for significance.

## 3 RESULTS

### 3.1 Estimating foliar traits using hyperspectral data

**Figure 2:**
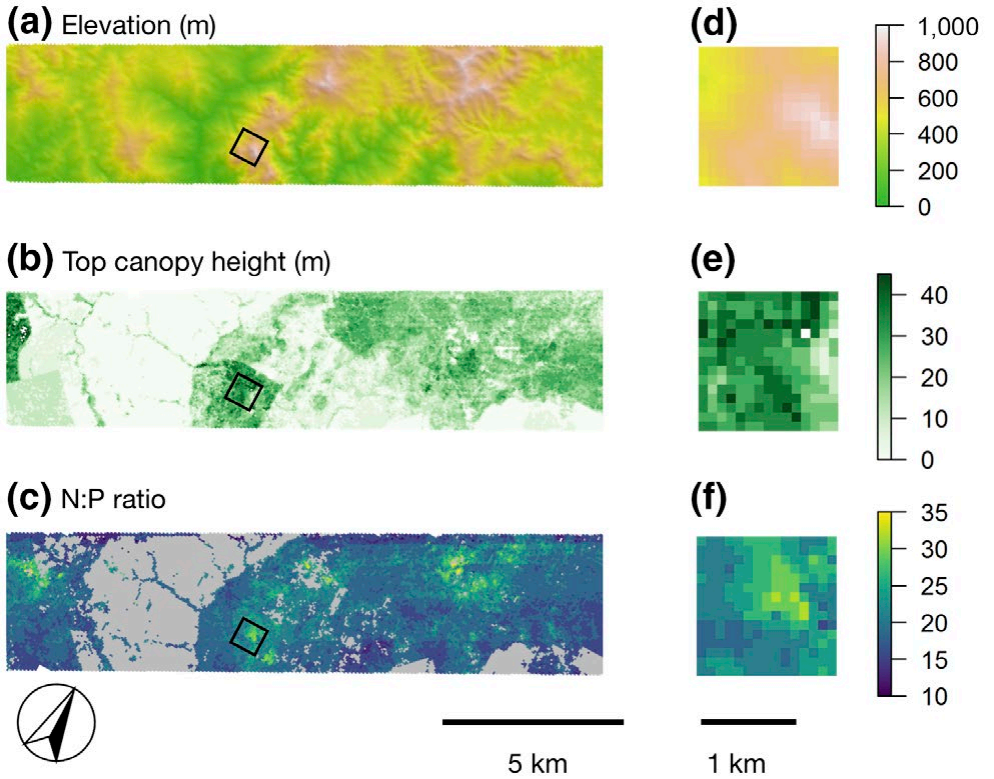
Variation in light detection and ranging measured (a) elevation and (b) top canopy height, and spectranomic estimates of (c) N:P ratio across the Stability of Altered Forest Ecosystems landscape at 100 × 100 m resolution. Non‐tree vegetation (<4 m in height) for which predictions were not made are shown as grey areas. Panels (d)–(f) show enlarged sections (black squares on the main panels), highlighting the role of elevation in shaping P limitation

The PLSR approach for estimating field measured foliar traits from crown‐level brightness‐normalized spectra performed satisfactorily, confirming the high performance of the spectranomic approach when cross‐validated by LOPO analysis (Chadwick & Asner, 2018). Out‐of‐sample cross‐validated *R* ^2^ of prediction values ranged from 41.5% to 12.8% (Table 1; validation plots are presented in Supporting Information S3). As anticipated, predictive performance was substantially lower using the more conservative LOTO cross‐validation, with *R* ^2^ of prediction values between 20.8% and 7.5% (Table 1). The best predictions were achieved for foliar [P] with lower predictive performance for N:P ratio, foliar [N] and SLA. Surprisingly, SLA exhibited the worst predictive performance despite being generally well predicted in other studies (Asner, Martin, et al., 2015; Doughty et al., 2017) and was not found to be meaningfully predicted by LOTO cross‐validation. These *R* ^2^ values were markedly lower than those ranging from 0.54 to 0.71 reported previously (Asner, Martin, et al., 2015). However, despite the low predictive performance of the PLSR models, the vast number of hyperspectral measurements across the landscape enabled us to assess sources of variation in plant traits induced by elevation and logging, which would not have been possible using the field plots alone due to the low number of samples available (Figure 2).

**Table 1:** Performance of partial least squares regression models to predict foliar traits from hyperspectral measurements, using commonly used leave‐one‐pixel‐out (LOPO) and the more conservative leave‐one‐tree‐out (LOTO) cross‐validation. The number of orthogonal spectral weighting components (n components) used in the final models is reported

Cross‐validation	Trait	*n* components	*R* ^2^	% RMSE
LOPO	Phosphorus concentration (%)^a^	9	41.5	37.5
	N:P ratio	8	36.6	24.2
	Total nitrogen concentration (%)^a^	7	32.0	30.1
	Specific leaf area (mm^2^/mg)^a^	3	12.8	24.3
LOTO	Phosphorus concentration (%)^a^	4	20.8	44.5
	N:P ratio	4	12.8	28.4
	Total nitrogen concentration (%)^a^	2	7.5	35.1
	Specific leaf area (mm^2^/mg)^a^	0	—	—

Note

*R* ^2^ of prediction values expresses the percentage of the total trait variation explained by the prediction. Percentage RMSE is the RMSE as a percentage of the mean trait value.

Abbreviation: RMSE, root mean squared error.

a PLSR models fitted to log transformed trait variables.

### 3.2 Spectranomic assessment of environmental effects on foliar traits

**Figure 3:**
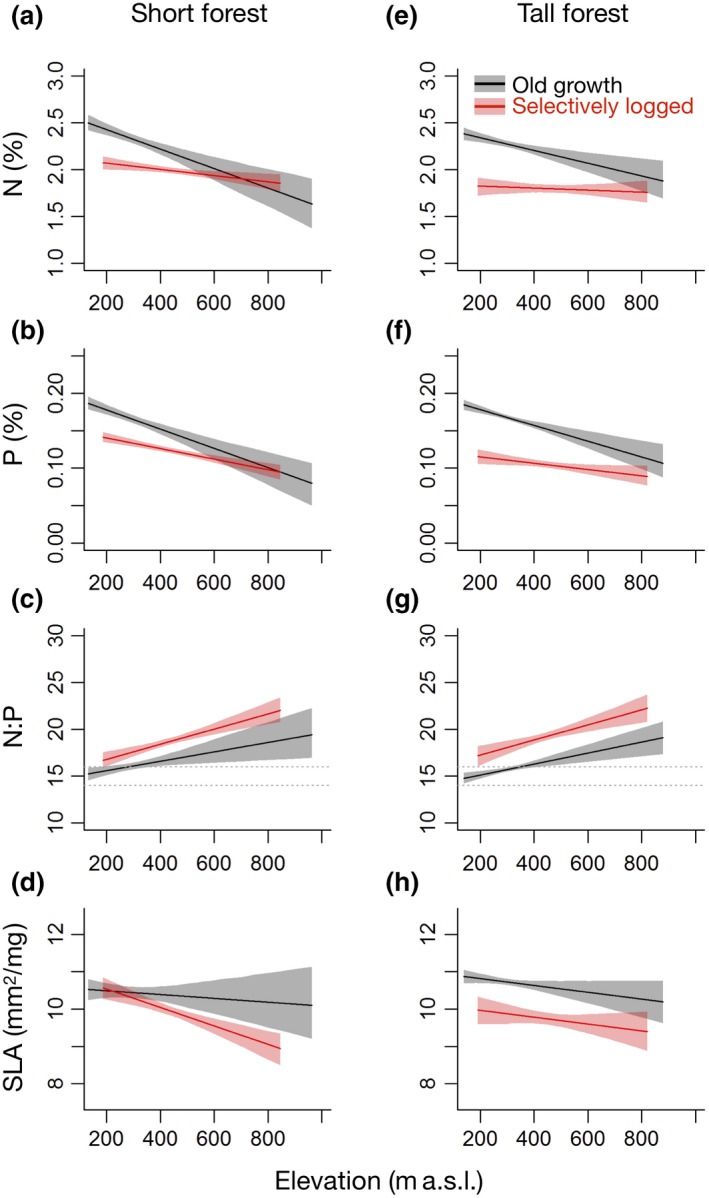
Comparison of spectranomic estimates of foliar traits in (a–d) short (10 m top canopy height) and (e–h) tall (30 m top canopy height) patches in logged (red) and old‐growth (black) forest landscapes. Relationships between elevation and (a, e) N concentration (per unit leaf mass); (b, f) P concentrations (per unit leaf mass); (c, g) N:P ratio; and (d, h) specific leaf area (SLA) are shown. Fitted lines show mean estimates from bootstrapped simultaneous autoregressive linear models and ribbons show the 95% confidence intervals for the relationships as estimated from 1,000 model iterations. The N:P range where N and P are co‐limiting (14–16) is shown with dashed grey lines

Our prediction that foliar nutrient concentrations and SLA would be lower in logged forest than in old‐growth forest due to nutrient limitation (H3) was supported: foliar [N] was estimated at 2.34% (95% CI = 2.29, 2.39) in old‐growth forest but was significantly lower at 1.82% (95% CI = 1.73, 1.91) in logged forest (Figure 3; Table 2). Similar relationships were found for foliar [P] which was estimated at 0.178% (95% CI = 0.174, 0.184) in old‐growth forest but only 0.115% (95% CI = 0.105, 0.125) in logged forest, and for SLA, which was estimated at 10.9 (95% CI = 10.7, 11.0) in old‐growth forest and 10.0 (95% CI = 9.6, 10.3) in logged forest (Figure 3).

**Table 2:** Model parameter estimates and confidence intervals for bootstrapped simultaneous autoregressive linear modelling of the relationships between the spectranomic estimates of canopy foliar traits and disturbance history (old‐growth vs. logged), top canopy height and elevation

	N (%)		P (%)		N:P		SLA (mm^2^/mg)	
	Mean	95% CI	Mean	95% CI	Mean	95% CI	Mean	95% CI
Reference	**2.34**	(2.29, 2.39)	**0.178**	(0.174, 0.184)	**15.1**	(14.7, 15.5)	**10.8**	(10.7, 10.9)
Logged	**−0.52**	(−0.68, −0.81)	**−0.063**	(−0.073, −0.052)	**2.14**	(1.05, 3.23)	**−0.85**	(−1.22, −0.49)
Elevation	**−0.11**	(−0.16, −0.05	**−0.016**	(−0.022, −0.010)	**0.92**	(0.45, 1.39)	−0.14	(−0.28, 0.01)
TCH	−0.03	(−0.06, 0.00)	0.000	(−0.003, 0.003)	−1.80	(−0.40, 0.03)	**0.12**	(0.03, 0.20)
Logged × elevation	**0.08**	(0.02, 0.16)	**0.010**	(0.001, 0.017)	0.33	(−0.40, 1.14)	−0.00	(−0.24, 0.24)
Logged × TCH	**−0.06**	(−0.11, −0.01)	**−0.010**	(−0.015, −0.003)	0.36	(−0.25, 0.95)	**−0.32**	(−0.52, −0.13)
Elevation × TCH	0.02	(−0.00, 0.05)	0.001	(−0.000, 0.005)	0.05	(−0.20, 0.30)	−0.02	(−0.09, 0.05)
Logged × elevation × TCH	−0.01	(−0.05, 0.02)	0.000	(−0.004, 0.004)	−0.06	(−0.45, 0.34)	0.11	(−0.02, 0.24)

Note

Parameters are reported relative to the reference level of 30 m tall, old‐growth forest at 200 m a.s.l. Coefficient estimates for top canopy height and elevation are standardized and correspond to changes of one standard deviation in the predictor; for top canopy height *SD* = 7.3 m; for elevation *SD* = 155 m. Confidence intervals and statistically significant coefficients, where estimates at the 95% confidence level did not overlap zero, are shown in bold. Abbreviation: SLA, specific leaf area.

We also predicted that foliar nutrient concentrations and SLA would be elevated in short patches due to shifts in functional composition with recent disturbance (H2) and this was identified in the logged landscape, where foliar [N] was 2.07% (95% CI = 2.00, 2.13) and foliar [P] was 0.140% (95% CI = 0.134, 0.147) for 10 m tall logged patches, significantly higher than the foliar nutrient concentrations of 30 m tall logged patches (Figure 3; Table 2). However, in old‐growth forest, no significant differences in foliar nutrient concentration were identified between short and tall forest patches (Figure 3; Table 2). Interestingly, foliar nutrient concentrations were more similar between short logged patches and short old‐growth patches than between tall logged patches and tall old‐growth patches (Figure 3). The differences in SLA between short logged patches and tall logged patches were small but significant, with SLA increased from 10.0 mm^2^/mg (see above) in tall logged patches to 10.5 mm^2^/mg (95% CI = 10.2, 10.8) in short logged patches (Figure 3; Table 2). Whereas there was a marginally significant decrease in SLA from 10.9 mm^2^/mg (see above) in tall old‐growth patches to 10.5 mm^2^/mg (95% CI = 10.2, 10.8) in short old‐growth patches (Figure 3; Table 2).

Higher foliar nutrient concentrations and SLA were predicted for low elevations due to the erosional and depositional processes that shape nutrient availability (H1). Significant negative relationships between foliar nutrient concentrations and elevation were identified in old‐growth forest, but this pattern was essentially absent in the logged forest. At 800 m a.s.l., foliar N and P concentrations were reduced to only 74.2% (mean = 1.76%; 95% CI = 1.65, 1.87) and 34.9% (mean = 0.120%; 95% CI = 0.106, 0.138) of the concentrations at 200 m a.s.l. respectively (Figure 3; Table 2). However, weaker elevational relationships in logged forest led to N and P concentrations of 96.4% and 77.8% of those at 200 m a.s.l. respectively (Figure 3; Table 2). This combination of strong elevational relationships in old‐growth forest and a weak relationships in logged forest meant that the large differences in foliar N and P concentrations at 200 m a.s.l. had practically disappeared by 800 m a.s.l. (Figure 3). For SLA, the relationship with elevation was generally weaker than for the foliar nutrient concentrations and was not found to be significant either alone or in interaction with TCH or disturbance history (Table 2).

Foliar N:P ratios were predicted to increase with both elevation and logging (H4) due to increasing phosphorus limitation. Indeed, N:P ratios increased significantly with elevation (Table 2); at 200 m a.s.l., the N:P ratios of old‐growth forest were 14.8, within the co‐limiting range (14–16), but above 286 m a.s.l. P was found to be limiting, with N:P ratios reaching 18.4 by 800 m a.s.l. (Figure 3). Logging also increased N:P ratios significantly (Table 2), by an average of 2.26 (95% CI = 1.80, 2.71) when compared with old‐growth forest, which was consistent across both short and tall forest patches (Figure 3).

### 3.3 Field‐assessed effects of elevation on foliar traits and soil nutrients

Field measurements of foliar traits (i.e. community‐weighted means) fell within the range of estimates produced by the spectranomic approach; 1.50%–2.20% for [N], 0.904%–1.643% for [P], 9.60–11.31 mm^2^/mg for SLA and 14.3–21.8 for N:P ratio. However, due to the small sample size (*n* = 8), there was no significant support for a relationship with elevation or logging for any trait, except some evidence of a weak negative trend in community‐weighted mean (CWM) foliar [P] and N:P ratio (Supporting Information S3). Similarly, total soil P showed some evidence of a weak negative trend with elevation, which (although not significant) suggested decreases of 65 mg/kg for each 100 m increase in elevation. Total soil N, as well as exchangeable soil N and P exhibited no relationships with elevation or logging (Supporting Information S4).

## 4 DISCUSSION

### 4.1 Trait variation associated with topography

Elevation was correlated with canopy foliar traits within the hilly landscapes of Sabah, with higher foliar nutrient concentrations and SLAs prevalent at low elevation. This provides support for hypothesis 1 that low elevation sites are more fertile than high elevation sites (Chadwick & Asner, 2016; Richardson et al., 2008). The reduction in foliar [P] at uphill sites was stronger than for foliar [N], leading to increases in N:P ratio uphill, supporting hypothesis 4. The N:P ratio exceeded 14–16, the range over which P is thought to be co‐limiting with N, across the majority of the study area, indicating increasing scarcity of P at uphill sites and a potential functional shift from acquisitive to conservative resource acquisition strategies (Mayor, Wright, & Turner, 2014; Porder et al., 2007; Tanner et al., 1998; Vitousek, 1984). These patterns were corroborated by similar trends identified through detailed field measurements of CWM foliar traits (Both et al., 2019; Supporting Information). However, despite suggestive trends, none of these CWM foliar traits exhibited significant relationships with elevation. This highlights the value of the spectranomic approach for upscaling detailed field surveys to reveal landscape‐scale patterns that would otherwise go undetected through traditional field methods (Asner, Martin, et al., 2015).

The elevational patterns observed are likely explained by weathering and the translocation of soil minerals from hill tops downslope (Quinton et al., 2010; Werner & Homeier, 2015). Rock‐derived soil nutrients are available at much higher concentrations in gullies than on nearby ridges and slopes, leading to corresponding increases in foliar nutrient concentrations downslope (Richardson et al., 2008; Werner & Homeier, 2015). A recent study using the spectranomic approach at the scale of individual trees in the Amazon found that foliar nutrient concentrations increase rapidly with gully depth, with stronger relationships for foliar [P] than for foliar [N], highlighting the importance of topography in shaping biogeochemical cycles and ultimately the availability of soil nutrients (Chadwick & Asner, 2016). Leaching of bases from high elevation sites in addition to the direct translocation of P may exacerbate P limitation by generating acidic conditions that reduce nutrient availability to trees (Tanner et al., 1998). This is supported by Both et al. (2019) who found lower soil pH in higher elevation logged plots when compared with lower elevation old‐growth plots. Finally, a phosphorus addition experiment by Liu et al. (2018) showed that ectomycorrhizal fungi, which only form associations with certain plant families, including the Dipterocarpaceae, are able to access complex forms of organic P with positive growth responses for associated tree species. The abundance of dipterocarps is much greater in lowland tropical forests (Kitayama, 1992; Pendry & Proctor, 1997) and is reduced by logging. If these trees are able to access increased quantities of organic P, they may increase foliar P availability at lower elevations, especially in old‐growth forest.

Elevational patterns in foliar [N] were weaker than those for foliar [P], resulting in increased N:P ratios on uphill sites. These patterns may be explained by the greater importance of topographic processes in determining the availability of soil P than N (Chadwick & Asner, 2016). While there is some evidence that labile soil P is translocated to a greater extent than soil N (Sharpley, 1985), it is more likely that fixation of N in combination with rainfall deposition counteracts the effects of any N translocation and denitrification so that N availability is less responsive to elevation than P availability (Brookshire, Gerber, Menge, & Hedin, 2012; Hilton, Galy, West, Hovius, & Roberts, 2013). We found no support for this hypothesis from our field measurements of exchangeable soil N and P, but a non‐significant negative elevational trend was identified for total P concentration. Nutrient availability fluctuates in a complex manner in response to abiotic and biotic conditions and instantaneous measures of nutrient availability may be inadequate to capture long‐term availability alone (Ghosh, Chatterjee, & Bremer, 2018). It is possible that total soil nutrient pools, including less accessible organic fractions and P bound to sesquioxides (Liu et al., 2018) provide better predictions of availability to tropical trees.

### 4.2 Differences in traits with canopy height in logged and old‐growth forest

After controlling for elevation, foliar nutrient concentrations and SLA were lower in logged forest than in old‐growth forest and lowest within tall patches within logged forest. This result supports our prediction that nutrient availability is reduced in logged forest leading to nutrient limitation as forests recover their biomass (Imai et al., 2012). Almost all of the SAFE landscape had been logged at least once, with some sites logged up to four times (Ewers et al., 2011). Each consecutive harvest is expected to reduce the pool size of mineral‐derived nutrients, including P (Imai et al., 2012), leading to progressive shifts towards functional traits that conserve scarce resources, such as low foliar nutrient concentrations and SLA (Carreño‐Rocabado et al., 2016; Li, Gu, Pang, Chen, & Liu, 2018). The greatest shift towards resource conservatism occurred in tall logged patches, where greater quantities of nutrients are doubtless required to support large quantities of additional leaves and biomass (Laclau, Bouillet, & Ranger, 2000). Carreño‐Rocabado et al. (2016) also found that as Bolivian forests recover, they diverge further from old‐growth functioning due to a greater dominance of palms. By contrast, at SAFE, short logged patches had higher nutrient concentrations and SLA than tall logged patches, despite being most intensely logged. A likely explanation is that fast‐growing pioneer species, specifically *Macaranga* and some *Mallotus* species with small seeds, low wood densities and large, thin, nutrient‐rich leaves were dominant in these heavily logged areas, as they are across the degraded forest landscapes of South East Asia (Primack & Lee, 1991; Riutta et al., 2018; Slik, 2005). And in the period following logging, while biomass remained low, light and nutrient availability were likely increased leading to functional shifts towards traits that favour resource acquisition (Baraloto et al., 2012; Brokaw, 1985; Gustafsson et al., 2016; Sterck, Markesteijn, Toledo, Schieving, & Poorter, 2014). Unexpectedly, however, the short logged patches had lower foliar nutrient concentrations and SLA than old‐growth forest. Our finding contrasts with that of Baraloto et al. (2012) who found higher foliar nutrient concentrations and SLA in a once‐logged forest and probably results from the repeated rounds of logging at SAFE.

### 4.3 Logging‐induced phosphorus limitation assessed by stoichiometry

Logging was associated with increased N:P ratios when compared to old‐growth forest, but these differences were not affected by canopy height. This provides some support for hypothesis 4, that is, that P limitation is exacerbated due to the extraction of timber from logged forests. Imai et al. (2012) calculate that P is extracted at a rate of 24 kg/ha during heavy selective logging activities in Borneo, exceeding the 11.8 kg/ha labile P in topsoil by more than double. Our finding supports this prediction, revealing that P availability is lower in logged forest as observed through a 35% reduction in canopy [P], with the greatest reductions in tall patches. An alternative explanation is that these changes are caused by the removal of tree species with higher than average concentrations of foliar P, such as Dipterocarps whose ectomycorrhizal symbionts can access organic P (Liu et al., 2018). Trees are able to reduce concentrations of metabolic P and nucleic acid P when growing on P‐poor soils, thereby increasing P‐use efficiency (Chadwick & Asner, 2016; Hidaka & Kitayama, 2011; Richardson et al., 2008), and the effect of logging appears to be small relative to the large natural variation in P availability caused by topography (Richardson et al., 2008). However, a 5 year manipulation experiment by Sayer and Tanner (2010) in Panama found that litter removal had little effect on P in soil, litter or leaves, indicating that foliar nutrient concentrations are remarkably resilient to relatively large changes in P inputs. They observed increases in litterfall in response to litter addition, arguing that the lack of a foliar P response may be caused by the ‘dilution effect’, whereby increased nutrient availability leads to increased production of leaves rather than increased foliar P concentration. This is supported by a shade‐house experiment in Panama where substantial variation in the growth response of pioneer species to P addition was not accompanied by changes in foliar P (Zalamea et al., 2016). The consequence of this variation is that community growth rates may not be strongly affected by P limitation because certain species exhibit high growth rates even when P is scarce (Turner et al., 2018; Zalamea et al., 2016). The higher N:P ratios and lower foliar nutrient concentrations in the logged forest at SAFE are suggestive of increased P limitation due to logging, but further experiments into the role of P limitation on growth are required before conclusions can be made regarding consequences for ecosystem function. Furthermore, it is possible that the differences observed between our sites may reflect broader dissimilarities in the underlying edaphic conditions, which cannot be ruled out by our study (Both et al., 2019).

### 4.4 Pixel‐level accuracy versus statistical power

The advantage of remote sensing lies in the massive number of measurements made, providing opportunities to map entire landscapes and increase statistical power. However, the spectranomic estimates of functional traits will never be as accurate as those made on the ground due to their indirect nature (Nunes et al., 2017). Indirect measurements, such as leaf traits measured through imaging spectrometry, contain measurement error arising as the residual error of statistical models used for trait prediction. This error is conveyed to trait predictions in a manner similar to regression to the mean, with large trait values reduced on average and small values increased, which is amplified when model accuracy is low. Therefore, when inference is sought, regarding the role of an environmental variable in determining leaf traits, the relationship will always be underestimated due to dampening of true trait values through the spectranomic approach. Field measurements by contrast provide a direct description of the precise traits that on aggregate measure ecosystem function. However, the effort required to sample the foliar traits of eight, one hectare plots were immense and, as we have demonstrated, it was not possible to fully explain the landscape processes important in shaping plot level differences from these measurements (Both et al., 2019). Despite the relatively low pixel‐level accuracy of our models, the predictions they produced provided thousands of virtual plots with which to test multiple hypotheses about how traits are shaped by landscape processes, including disturbance. This allowed us to access the statistical power of a large observational data set and detect patterns that would otherwise go undetected using field data alone (Asner & Martin, 2016b; Jucker et al., 2018; Schneider et al., 2017).

The low predictive performance of our models, as assessed through leave‐one‐tree‐out cross validation, suggests there was insufficient spectral information to describe trait variation at the scale of individual trees, as we had hoped. Instead, our predictions likely reflect patterns detectable through spectral differences at the plot scale, which has more broadly been shown to be successful (Asner, Martin, Ford, Metcalfe, & Liddell, 2009; Asner et al., 2011; 1975). The low variance in community‐weighted SLA among the plots (Supporting Information S2) perhaps explains why SLA was predicted with low precision compared with other studies that have found that SLA is well predicted (Asner, Martin, et al., 2015; Asner et al., 2011; Doughty et al., 2017). Better predictive performance has been achieved by predicting leaf traits from spectra measured under laboratory conditions (Doughty et al., 2017; Nunes et al., 2017). As anticipated, predictive models based on airborne hyperspectral measurements are less accurate, because tree‐level spectral signatures combine light reflected by overlapping and adjacent crowns, in addition to canopy structural properties, that further modulate the signal (Asner et al., 2011, 2017; Doughty et al., 2017). Assessments of predictive performance are likely to be inflated when field samples are collected in a clustered manner, which is not accounted for during analysis (Rocha, Groen, Skidmore, Darvishzadeh, & Willemen, 2018), particularly if both trait and spectral variation is small within clusters relative to between clusters (e.g. Chadwick & Asner, 2016). Although our data were also collected in a clustered manner, we have attempted to account for this using leave‐one‐tree‐out cross validation, which ultimately produced more conservative estimates of model accuracy. Furthermore, we have demonstrated that within plot trait variation is almost as large as between plot variation (see Supporting Information S2), which is likely to have contributed to lower predictive performance.

### 4.5 The impacts of logging on biogeochemical cycling

Spectranomic analysis of hyperspectral imagery revealed widespread changes in the foliar traits of tropical forests resulting from logging, with important implications for biogeochemical cycling. We found that foliar nutrient concentrations and SLA were lower in logged forests than in old‐growth forests in Sabah, after controlling for landscape elevation and TCH. Decreased foliar nutrient concentrations in logged forests are concerning because they demonstrate that repeat cycles of logging on infertile tropical soils produce forests that are functionally distinct from the old‐growth forests they replace (Imai et al., 2012; Markewitz, Davidson, Moutinho, & Nepstad, 2004). Given that the P content of soils is a strong determinant of community composition (Condit, Engelbrecht, Pino, Pérez, & Turner, 2013), removal of significant quantities of P through repeated timber extraction could lead to major changes in tree species composition in logged tropical forests, with major implications for productivity and other ecosystem processes (Zalamea et al., 2016; cf. Turner et al., 2018).
