# Supplementary material for: Imaging spectroscopy reveals the effects of topography and logging on the leaf chemistry of tropical forest canopy trees
Source: Glob Chang Biol. 2019 Dec 17;26(2):989–1002. doi: 10.1111/gcb.14903 (PMC7027875; doi:10.1111/gcb.14903)
Supplement: Supplementary file 1 [file GCB-26-989-s001.docx]

## Supplementary information

### S1. Tree crown polygon alignment method

The field measured crown positions were converted to spatial polygons and aligned manually to the LiDAR canopy height models in QGIS (Fig. S1.1a). Any trees which had field measured canopy heights which poorly matched the LiDAR canopy heights were excluded. Where possible, any trees that were overtopped by a taller tree were cropped only to the crown section observable from above, otherwise these too were excluded. In addition we allocated confidence scores to each resulting polygon and ultimately only retained polygons with medium or high confidence scores (Fig. S1.1b).


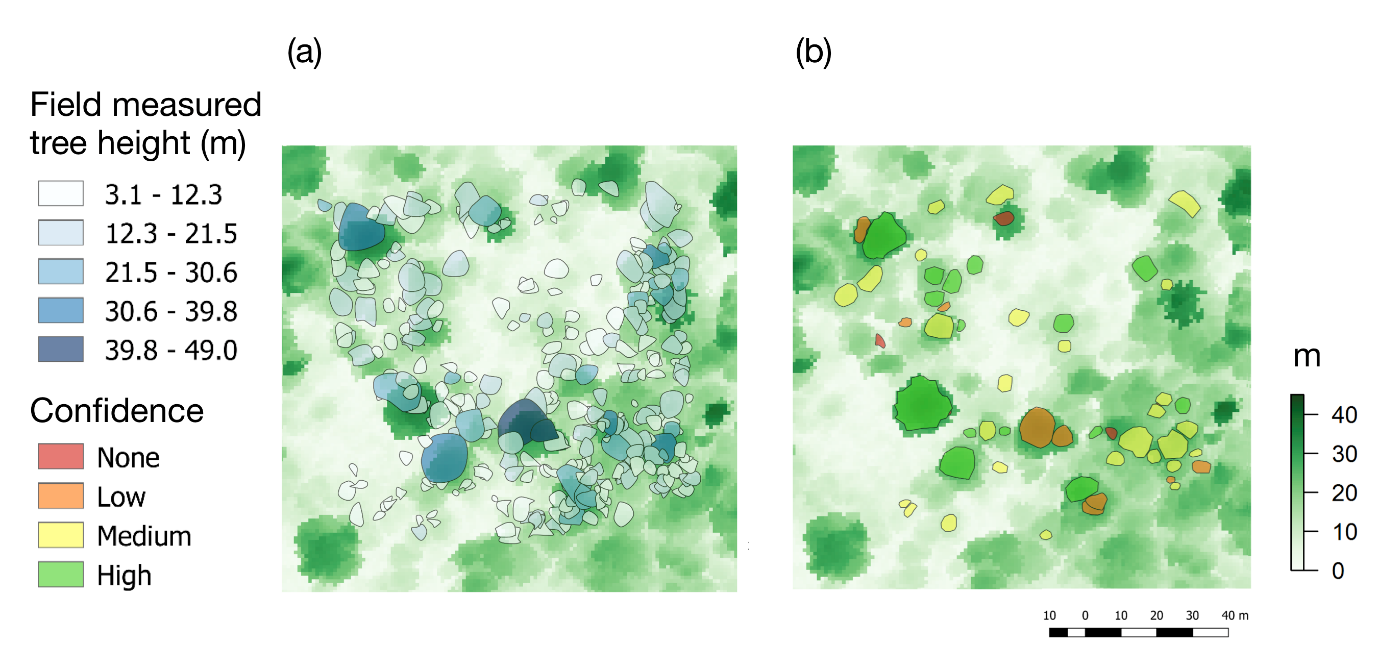


**Figure S1.1.** The alignment of field measured tree crown positions to the LiDAR measured canopy height model. (a) Field measured tree crown positions (a) prior to and (b) after alignment.

###

### S2. Trait distributions by plot

A subset of the trees whose traits were sampled were used to train the partial least squares regression (PLSR) model to predict traits from hyperspectral data. Trait sampling, as described in the methods section, was implemented to ensure that the distribution of traits at the community level, within each plot, was thoroughly captured. However, because hyperspectral imagery predominantly captures reflectance data from the canopy surface, only trees whose crowns, or parts of crowns, were observed conspicuously in the LiDAR canopy height model were included in the predictive model. Furthermore, only traits measured on sunlit branches were used as it was assumed that the spectral signature observed by the spectrometer is dominated by sunlit leaves. Subsetting like this potentially introduces bias; however plot-level mean and variances of traits closely matched those observed from all sunlit and indeed shaded leaves (Figure S2.1-4).


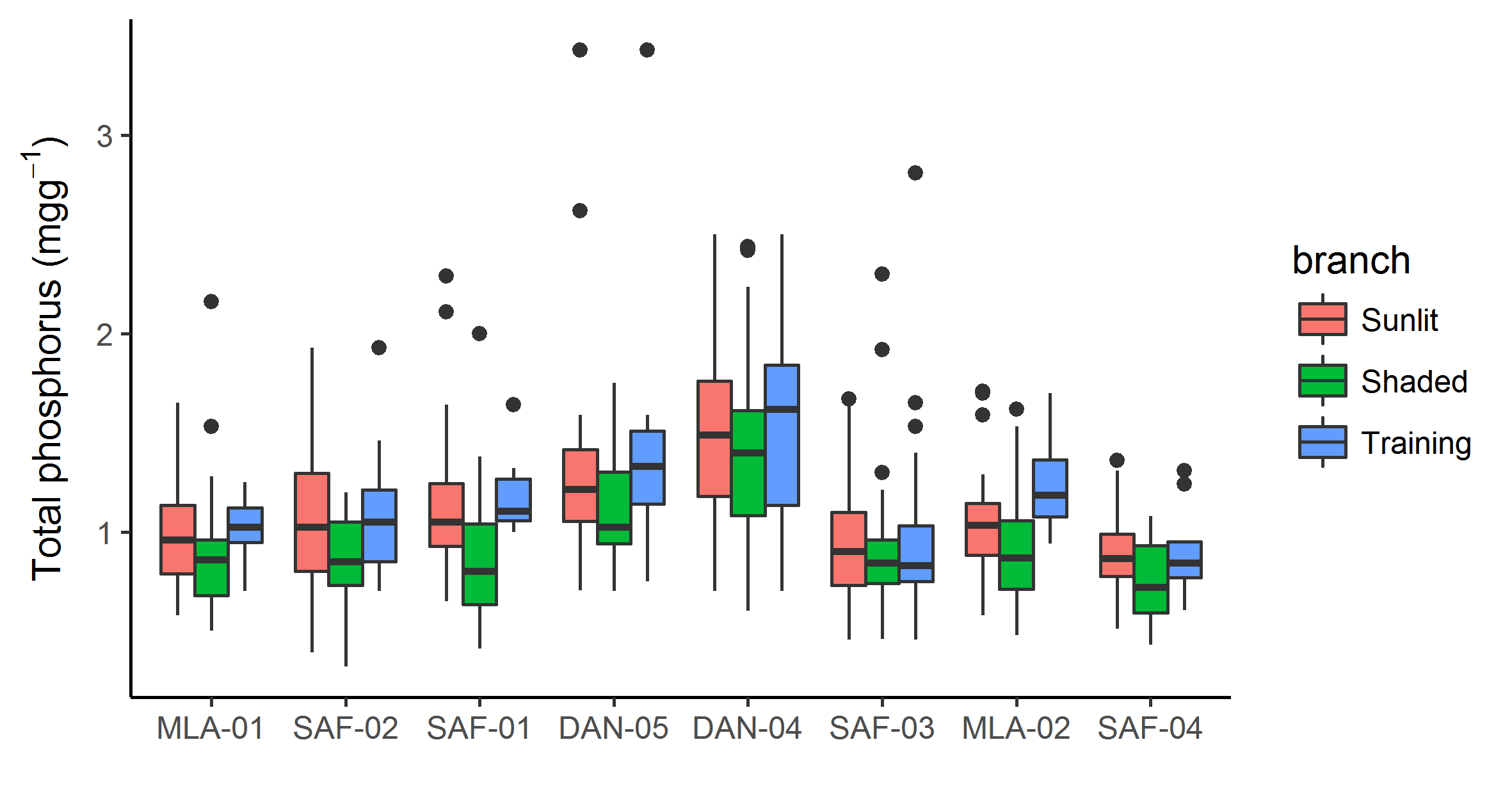


**Figure S2.1.** Plot level trait distributions of total foliar phosphorus concentrations per unit mass (mg g^-1^) for leaves collected from sunlit and shaded branches in Danum (DAN-04 and DAN-05), Maliau (MLA-01 and MLA-02), and SAFE (SAF-01, SAF-02, SAF-03 and SAF-04). These are compared with the subset of sunlit leaves collected from canopy trees used to train the PLSR models to predict traits from hyperspectral data.


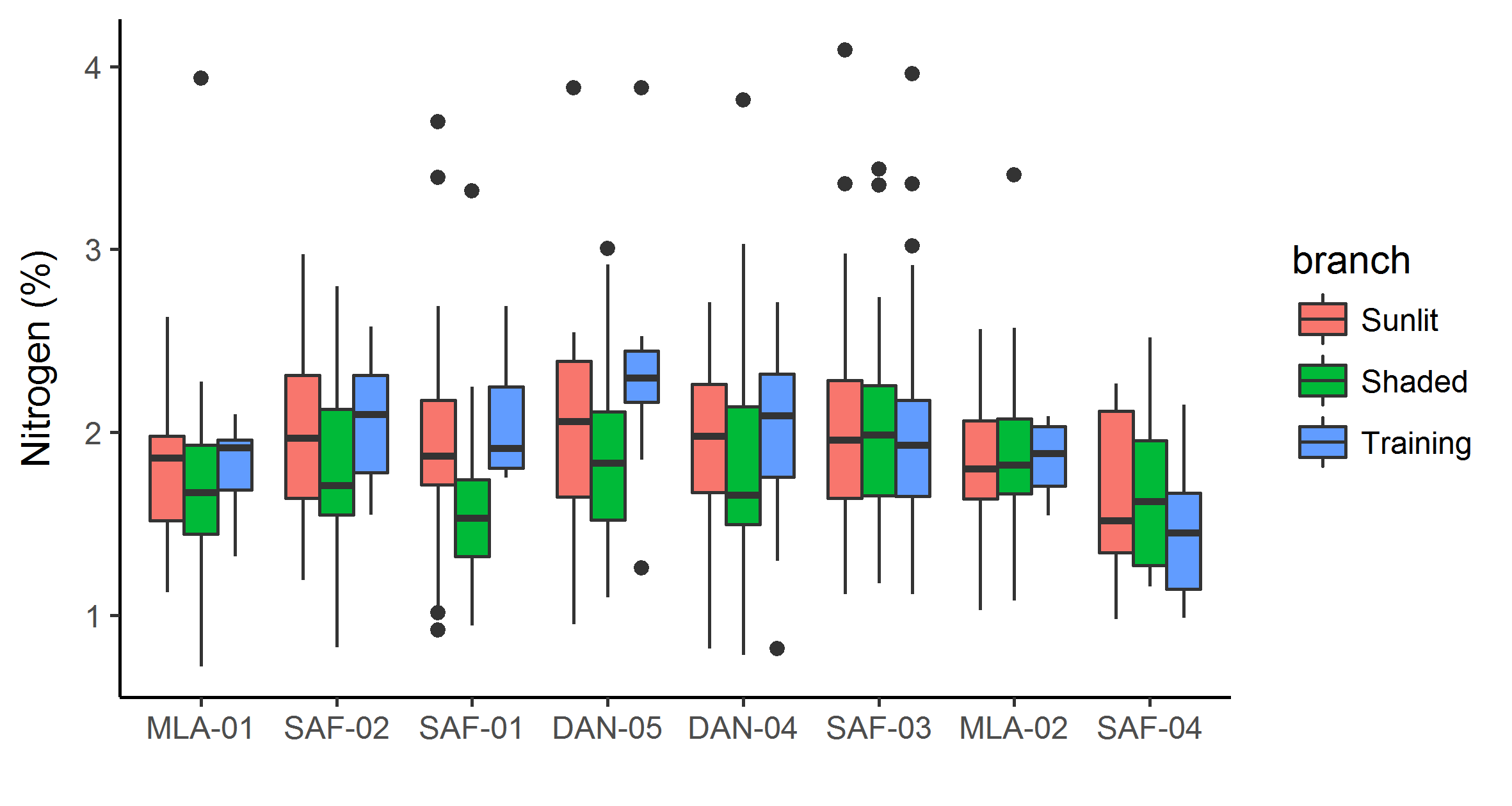


**Figure S2.2.** Plot level trait distributions of nitrogen concentrations per unit mass (%) for leaves collected from sunlit and shaded branches in Danum Danum (DAN-04 and DAN-05), Maliau (MLA-01 and MLA-02), and SAFE (SAF-01, SAF-02, SAF-03 and SAF-04). These are compared with the subset of sunlit leaves collected from canopy trees used to train the PLSR models to predict traits from hyperspectral data.


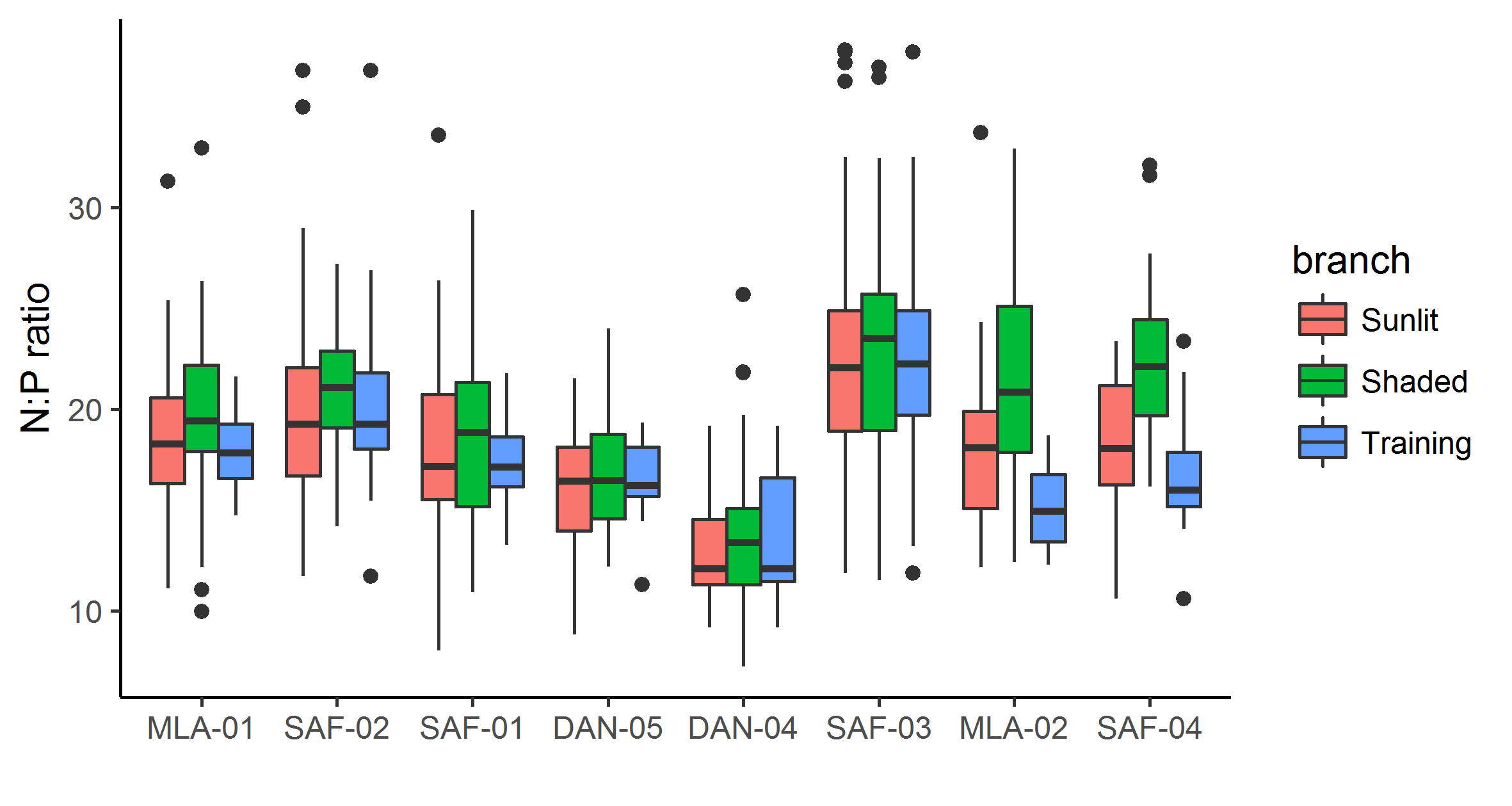


**Figure S2.3.** Plot level trait distributions of N:P ratio for leaves collected from sunlit and shaded branches in Danum (DAN-04 and DAN-05), Maliau (MLA-01 and MLA-02), and SAFE (SAF-01, SAF-02, SAF-03 and SAF-04). These are compared with the subset of sunlit leaves collected from canopy trees used to train the PLSR models to predict traits from hyperspectral data.


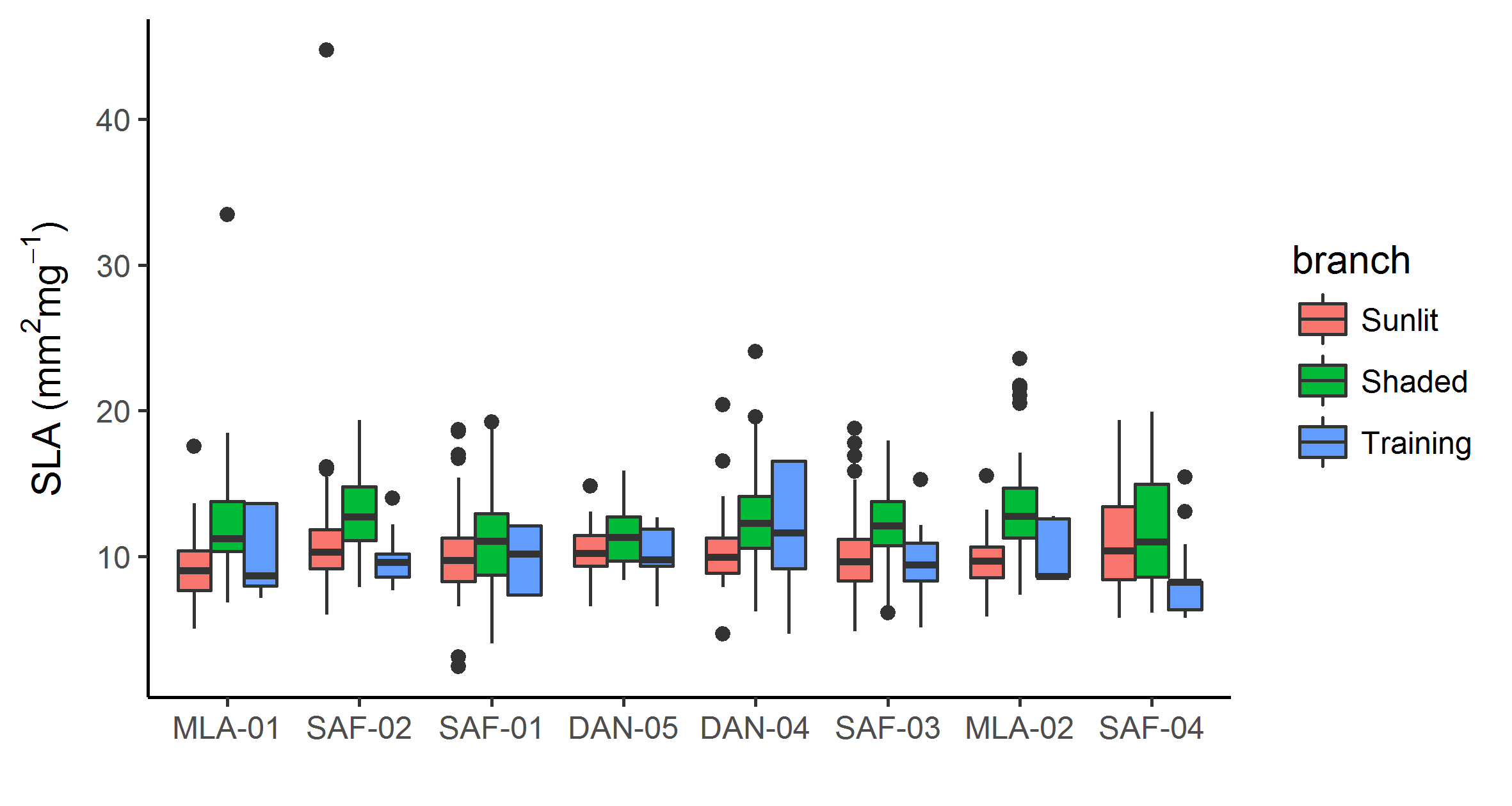


**Figure S2.4.** Plot level trait distributions of specific leaf area (mm^2^ mg^-1^) for leaves collected from sunlit and shaded branches in Danum (DAN-04 and DAN-05), Maliau (MLA-01 and MLA-02), and SAFE (SAF-01, SAF-02, SAF-03 and SAF-04). These are compared with the subset of sunlit leaves collected from canopy trees used to train the PLSR models to predict traits from hyperspectral data.

### S3. Field measured community-weighted mean foliar traits

Community-weighted mean traits were calculated at the 1-ha plot level by combining the field-based trait measurements with the forest inventory data as:

$\bar{x}=\sum_{i=1}^{n} x_{i}w_{i}$ (1)

Where *x* was the mean trait value and *w* was the species-specific weighting for the *i^th^* of *n* species for which trait data were collected*.* Species level mean trait values (*x_i_*) were calculated at the plot level by averaging trait measurements from sunlit leaves. Species weightings (*w_i_*) were calculated as the proportion of total plot level basal area comprised by that species.


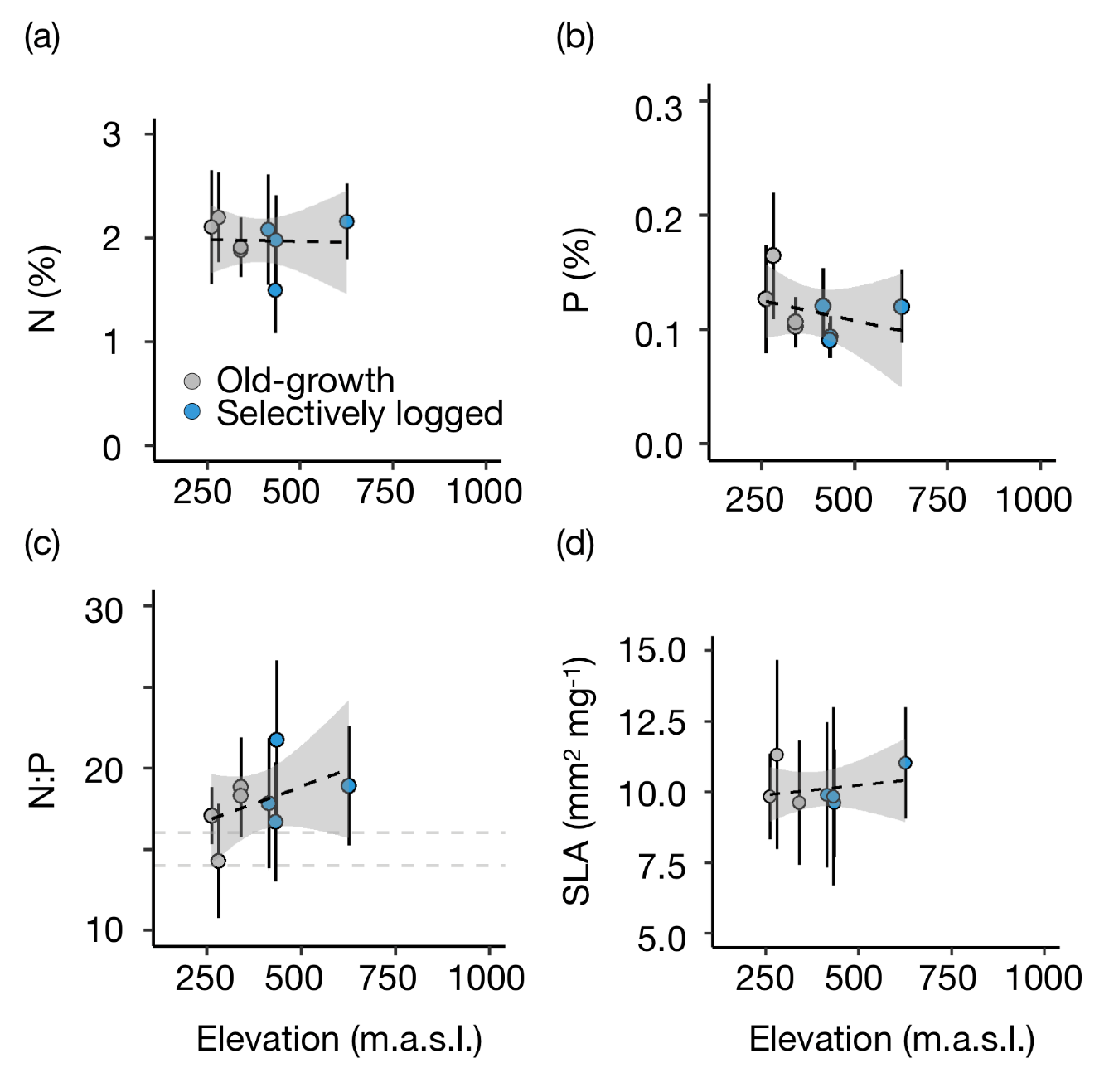


**Figure S3.** Relationships between elevation and field measured community weighted mean (CWM) foliar traits in old-growth (grey) and selectively logged (blue) forests. CWM (a) N concentration per unit mass; (b) P concentrations per unit mass; (c) N:P ratio; and (d) specific leaf area for the eight 1 ha plots are shown. Error bars show community-weighted standard deviations, and dashed lines and shaded ribbons show the fitted linear models (which were not significant for all four traits) and the 95% confidence intervals for the relationships between elevation and the traits. The N:P range where both nitrogen and phosphorus are co-limiting (14-16) is shown with dashed grey lines.

### S4. Field measured soil nutrient availability

Total and ion-exchangeable nutrient pools in soil were measured; the former provides an estimate of the potential for soils to supply plants with nutrients, while the latter provides information on the immediate availability of nutrients to plants (e.g. Ghosh *et al.* 2018). Two soil surface cores (to 10 cm depth) were taken randomly from each plot in 2014-15 and analysed for total P and N following Quesada et al. (2010, 2012). Exchangeable NO_3_^-^ and NH_4_^+^ were measured using *in situ* ion exchange membranes (PRSTM Probes, Western AG, Saskatoon, Canada). Four pairs of probes (cation and anion) were installed vertically (to 10 cm depth) at the corners of three 50 cm × 50 cm quadrats within each of the subplots used in the supplemented random sampling of the foliar traits, as defined in the *Foliar trait measurement* section below. Probes were collected after two weeks, washed with distilled water and sent for analysis. The four probe pairs for each quadrat were lumped prior to elution with 0.5M HCl for 1 hour, yielding a total of 72 samples. NO_3_^-^ and NH_4_ were measured colorimetrically using automated flow injection analysis.


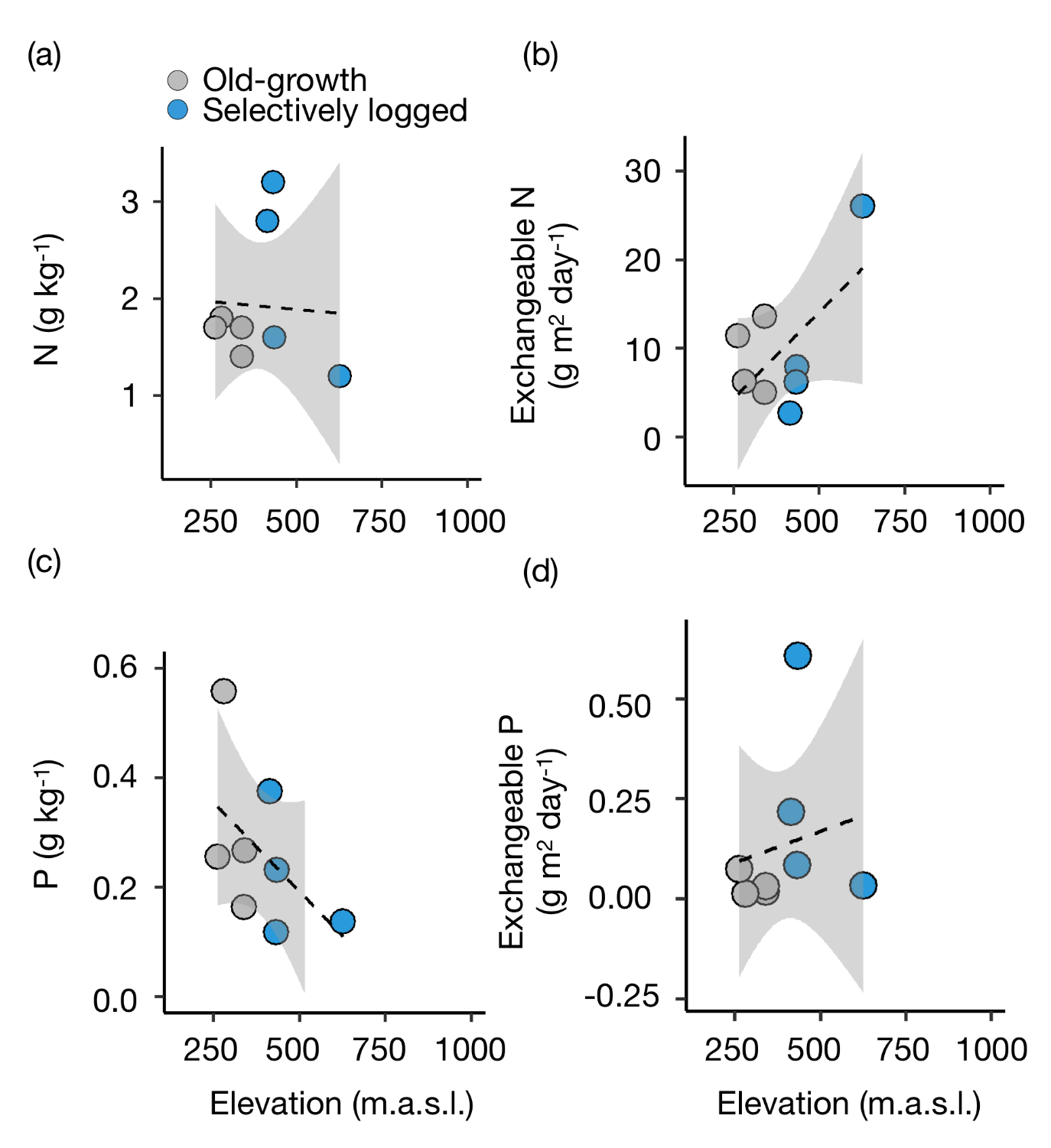


**Figure S4.** The relationship between elevation and field measured (a) total soil nitrogen, (b) exchangeable soil nitrogen (nitrate + ammonium), (b) total soil phosphorus, and (d) exchangeable soil phosphorus. Old-growth and selectively logged forest plots are shown in grey and blue respectively. Dashed lines and shaded ribbons show the fitted linear models (which were not significant for all four traits) and 95% confidence between for the relationship between elevation and soil nutrients.
